# Temporomandibular disorder confounders in motor vehicle accident patients

**DOI:** 10.22514/jofph.2025.014

**Published:** 2025-03-12

**Authors:** Xiang Li, Vandana Singh, Camila Pacheco-Pereira, Reid Friesen

**Affiliations:** ^1^Mike Petryk School of Dentistry, University of Alberta, Edmonton, AB T6G 1C9, Canada; ^2^Private Practice in Edmonton, Edmonton, AB T6G 1C9, Canada

**Keywords:** Temporomandibular disorders, Traffic accidents, Motor vehicle accidents, Whiplash injuries, Cone-beam computed tomography, Diagnostic errors, Orofacial pain, Temporomandibular joint

## Abstract

**Background**: Motor vehicle accidents (MVA) are associated with the onset of temporomandibular disorder (TMD) symptoms. However, diagnosing TMD-related pain is challenging due to various entities that can refer pain to the region. This study aims to identify prevalent radiographic confounders to pain diagnosis in MVA patients who were subsequently referred for temporomandibular joint imaging using cone-beam computed tomography (CBCT) by comparing these patients to a cohort of patients without MVA history. **Methods**: CBCTs of 738 temporomandibular joints were reviewed, with cases stratified by MVA history. This research explored the demographics and calculated the prevalence of radiographic confounders (RC) in each category, comparing the findings for both groups. The chi-square test was used to assess statistical significance. **Results**: Patients in the MVA cohort (n = 151, mean age = 41.3 years, S.D (Standard Deviation) = 13.3 years) averaged 1.10 confounders/patient compared to a significantly lower 0.68 confounders/patient in the non-MVA cohort (n = 218, mean age = 33.6 years, S.D = 18.2 years). The most frequently identified RCs include sinus pathologies (39.1% (MVA) *vs.* 28.0% (non-MVA), *p* = 0.025) and endodontic lesions (22.5% (MVA) *vs.*10.1% (non-MVA), *p *= 0.001). **Conclusions**: Clinicians must be vigilant about confounders when managing patients suspected of TMD. We recommend patients undergo a complete dental evaluation before being referred to a specialist to avoid unnecessary medical costs and treatment delays.

## 1. Introduction

Temporomandibular disorders (TMD) are a group of conditions characterized by pain or dysfunction of the temporomandibular joint (TMJ) and its associated structures [1, 2]. TMD is estimated to affect 5%–12% of the population [2]. Patients suffering from TMD may experience considerable morbidity, which can negatively impact their quality of life. The most reported signs and symptoms include pain near the TMJ area aggravated by function, jaw pain, TMJ noises, limited mobility and headaches [1, 2]. TMD is considered the most common non-odontogenic cause of orofacial pain, and its etiology is multifactorial [3].

The primary modalities of establishing a preliminary differential diagnosis are obtaining a detailed history and performing a clinical examination [3, 4]. TMD symptoms can often suggest possible causes of the patient’s complaints. For example, crepitus can suggest osteoarthritic changes in the joint. However, additional advanced imaging is often required to evaluate the joint components to confirm or further delineate the probable cause of the patient’s presenting symptoms. Traditional 2-dimensional imaging modalities often have limited diagnostic value due to limited visualization of areas of interest, distortions, and superimpositions [5]. Meanwhile, cone-beam computed tomography (CBCT) provides a cost-efficient, three-dimensional (3D) assessment of the osseous components of the TMJ without the aforementioned limitations. At the same time, CBCT enables clinicians to assess surrounding anatomical structures to exclude coexisting pathologies resembling TMD symptoms. However, CBCT’s primary drawback is its limited soft tissue contrast [5]; hence, magnetic resonance imaging (MRI) is recommended for soft tissue evaluation (including disc displacement), while cone-beam computed tomography (CBCT) is primarily used for osseous assessment.

TMD onset has been correlated with a history of motor vehicle accidents (MVA), with 14%–37.5% of the patients who experienced whiplash incidents estimated to develop TMD [6]. A recent prospective study using MRI imaging and clinical interviews concluded that patients subjected to whiplash incidents suffer from significantly more TMD symptoms than the control group both immediately and up to 15 years after the incident [7]. This relationship between MVA and TMD has important legal and clinical implications. In Canada alone, there were 89,787 MVA involving 118,853 people in 2022 [8].

MVA cases can result in significant court-awarded damages. For instance, in the case of Russell V. Turcott, the Canadian Court of Queen’s Bench awarded a six-figure settlement, in which TMD-related pain was an important consideration [9]. Costs associated with the diagnosis, treatment and ongoing maintenance treatment of TMD and other concurrent orofacial injuries can still be substantial for at-fault drivers or their vehicle insurers [10]. In the United States, it was found that insurance claims for TMD patients were on average double the total cost of non-TMD patients, indicating a significant increase in medical expenses after a TMD diagnosis [11].

Pain complaints arising from the TMJ present a diagnostic challenge due to various possible entities of orofacial pain that can exhibit similar clinical presentations [12]. Notably, 75% of orofacial pain can be attributed to odontogenic origins [13]. In the current literature pool, there is no comprehensive data on the presence of radiographic confounders (RC) that can mimic TMD-related pain in patients who were referred for a CBCT investigation of the TMJ after a MVA. Therefore, the two-fold aim of this study is to bridge the knowledge gap by identifying the most prevalent RCs in this patient cohort and to compare the findings to patients without this history. Ultimately, our goal is to raise awareness among clinicians and legal experts on the multiple sources of orofacial pain that can present similarly to TMJ-related pain and their association with MVA.

## 2. Materials and methods

This retrospective study obtained approval from the Human Research Ethics Board at the University of Alberta under Pro00129205.

We first obtained CBCT scans and the corresponding oral and maxillofacial radiology (OMR) reports from all the patients who underwent a TMJ scan at the University of Alberta and at two local private practices from July 2020 to June 2023. The CBCT scans were obtained exclusively for clinical diagnostic purposes, their justification and prescription followed the clinical examination, and they were not acquired for research purposes. Board-Certified Oral and Maxillofacial Radiologists reviewed all reports. CBCTs taken for indications other than for TMJ investigation were excluded from the study.

We stratified our pool of cases to either the non-MVA group or the MVA group based on the patient’s history of MVA as indicated in their electronic health records, CBCT requisition and referral forms. Only motor vehicle accidents (MVAs) that occurred within two years of the scan were included in the MVA group, with CBCT imaging completed within this two-year time frame. However, exact time intervals between the MVA and the CBCT scan could not be obtained due to challenges retrieving this information from the available data. All patients underwent comprehensive assessments by medical doctors and allied healthcare professionals, such as physiotherapists and chiropractors before referral to the Oral Medicine clinics. These evaluations ensured that patients were free from significant facial fractures or severe neck injuries, which could confound TMJ-related symptoms. During these assessments, patients were specifically assessed regarding any facial fractures or neck injuries. This screening process ensured the exclusion of patients whose pain or dysfunction could be directly attributed to acute conditions such as neck or facial fractures.

Data on demographics, image acquisition parameters and clinical information were collected from each patient’s OMR reports and requisition forms. Subsequently, each report was carefully reviewed to identify any potential RCs present in each CBCT scan.

The prevalence of each category of RC was calculated and compared between groups. The chi-square test was used to test if the distribution of the results differed significantly from the two population samples. Statistical significance is defined when the *p* value is less than 0.05. Data analysis was performed using Statistical Package for Social Sciences (SPSS, version 23, Armonk, NY, USA).

## 3. Results

Out of the 369 CBCT images acquired, representing 738 TMJs, 151 (40.9%) of the patients had a previous history of MVA involvement within the past 24 months while 218 (59.1%) of the patients had not.

Patient demographic variables and CBCT scan field of view (FOV) used for the image acquisitions are summarized in Table 1, with the mean age of the MVA group and the non-MVA group being 41.3 years (standard deviation (S.D) of 13.3 years) and 33.6 years (S.D 18.2) respectively. Large FOV CBCT scans accounted for most of the scans (75.1%) with medium FOV (14.1%) and small FOV restricted to the TMJ (10.8%) making up the remainder of the scans.

**Table 1.** Patient demographics stratified by history of motor vehicle accidents (MVA).

		MVA Group		Non-MVA Group		Total
		N	%	N	%	N
Gender						
	Male	56	45.5	67	54.5	123
	Female	95	38.6	151	61.4	246
CBCT FOV						
	Small	0	0.0	40	100.0	40
	Medium	15	28.8	37	71.2	52
	Large	136	49.1	141	50.9	277
Age of Patient		Mean = 41.3 yr, S.D = 13.3 yr		Mean = 33.6 yr, S.D = 18.2 yr		Mean = 36.4 yr, S.D = 17.1 yr

*Abbreviations: MVA: motor vehicle accident; S.D: standard deviation; CBCT: cone-beam computed tomography; FOV: Field of View.*

Table 2 depicts the TMJ provisional diagnosis from CBCT scans stratified according to whether the patient had an MVA history. Notably, the non-MVA group had a significantly (*p *= 0.02) greater number of diagnoses of normal anatomy (15.1%) compared to the MVA group (7.3%). Meanwhile, the non-MVA group exhibited a significantly (*p* = 0.04) greater number of idiopathic juvenile arthritis diagnoses (2.8%) compared to the MVA group (0.0%). Furthermore, diagnosis of TMJ functional remodelling and degenerative joint disease (active and non-active) were all more prevalent in the MVA group compared to the non-MVA patient cohorts.

**Table 2.** Provisional diagnosis of TMJ findings stratified by the presence of MVA history.

TMJ-CBCT Provisional Diagnosis	Non-MVA Group		MVA Group		Total		χ^2^ value	*p* value
	N	%	N	%	N	%		
None	33	15.1	11	7.3	44	11.9	5.24	0.02*
TMJ Remodelling	55	25.2	49	32.4	104	28.2	2.30	0.13
Degenerative Changes (non-active DJD)	43	19.7	35	23.2	78	21.1	0.64	0.42
Degenerative Joint Disease	69	31.7	52	34.4	121	32.8	0.31	0.58
Idiopathic Juvenile Arthritis	6	2.8	0	0.0	6	1.6	4.23	0.04*
Others	4	1.8	3	2.0	7	1.9	0.01	0.92
Progressive Condylar Resorption	8	3.7	1	0.7	9	2.4	3.39	0.07
Total	218	100.0	151	100.0	369	100.0		

*Chi-Square Test: Not significant: p > 0.05, Significant: *p < 0.05. Abbreviations: TMJ: temporomandibular joint; CBCT: cone-beam computed tomography; MVA: motor vehicle accident; DJD: degenerative joint disease.

Table 3 summarizes the prevalence of each category of RCs based on the patient’s history of MVA. The identified radiographic confounders in post-MVA patients present various types of pain that can overlap with TMD symptoms. Impacted dentition can lead to trismus and localized jaw pain, sinus pathology often results in midfacial pain resembling masseter muscle discomfort, and periapical pathology may cause referred pain to the jaw [14, 15, 16]. Additionally, an elongated stylohyoid process can contribute to neck pain, while previously fractured condyles may lead to recurrent joint pain, all of which may be misinterpreted as TMD-related pain [17, 18, 19]. From our results, we have determined that the patients in the non-MVA group had an average of 0.68 RC per patient compared to a statistically significant higher average of 1.10 RCs per patient for the MVA group. Most notably, the prevalence of sinus pathologies (*p *= 0.025) and endodontic lesions (*p *= 0.001) were both significantly higher in the MVA cohort compared to the non-MVA group of patients. Sinus pathologies include sinusitis, moderate to severe mucosal thickening (including mucositis with acute inflammation features such as air-fluid levels) and cysts. While these were all recorded, it is important to note that not all sinus pathology invariably presents with pain symptoms. Other more commonly found RCs whose prevalence trended higher in the MVA group included impacted dentition (16.6% (MVA) *vs.* 10.1% (non-MVA)) and root fractures/retained root tips (8.6% (MVA) *vs.* 4.1% (non-MVA)).

**Table 3.** Radiographic confounders of TMJ CBCT scans by presence of MVA history.

Variables		Non-MVA		MVA		Total		χ^2^ value; *df* = 1	*p* value
		N	%	N	%	N	%		
Impacted dentition									
	No	196	89.9	126	83.4	322	87.3	3.35	0.067
	Yes	22	10.1	25	16.6	47	12.7		
Sinus Pathology									
	No	157	72	92	60.9	249	67.5	5.00	0.025*
	Yes	61	28	59	39.1	120	32.5		
Periapical pathology									
	No	196	89.9	117	77.5	313	84.8	10.70	0.001*
	Yes	22	10.1	34	22.5	56	15.2		
Previous traumatic condylar fracture									
	No	216	99.1	151	100.0	367	99.5	1.39	0.238
	Yes	2	0.9	0	0.0	2	0.5		
Root Fracture and residual root tips									
	No	209	95.9	138	91.4	347	94.0	3.20	0.074
	Yes	9	4.1	13	8.6	22	6.0		
Elongated/Calcified Stylohyoid process									
	No	196	89.9	139	92.1	335	90.8	0.49	0.484
	Yes	22	10.1	12	7.9	34	9.2		
Other soft tissue calcifications									
	No	217	99.5	150	99.3	367	99.5	0.07	0.793
	Yes	1	0.5	1	0.7	2	0.5		

*Chi-Square Test: Not significant: p > 0.05, Significant: *p < 0.05. Abbreviations: TMJ: temporomandibular joint; MVA: motor vehicle accident; CBCT: cone-beam computed tomography; df: degree of freedom.

## 4. Discussion

MVA is a public health concern worldwide, and it is associated with the presence of chronic pain and psychiatric disorders including post-traumatic stress and anxiety disorders [20, 21]. In the head and neck region, MVAs can cause traumatic injuries to the facial skeleton, as well as damage to the dental-alveolar complex, and contribute to the development of TMD [22, 23, 24]. The World Health Organization estimates the annual total economic burden related to MVA injuries to be US$ 1.8 trillion globally [25].

This is the first study to use CBCT to examine the presence of radiographic confounders in the oral and maxillofacial complex of patients presenting with pain and a history of MVA. These 3D scans are routinely used by oral medicine specialists or oral surgeons for assessing patients presenting signs and symptoms of TMD during clinical examination. We have found a considerably greater average number of RCs in the MVA cohort compared to the non-MVA cohort. The prevalence of sinus pathologies and endodontic lesions were both significantly greater in the MVA cohort (11.1% and 12.4% higher, respectively) and the prevalence of impacted dentition and retained root tips/root fractures both trended higher in the MVA group (6.5% and 4.5% higher, respectively). While sinus pathologies, such as sinusitis, mucosal thickening and cysts, were identified in the CBCT scans, not all of these conditions may be directly associated with the patient’s presenting pain [26]. Due to the retrospective study design, we cannot establish a direct causal link between the incidental radiographic dental findings and the MVA itself, nor was this the goal of our study. Instead, the importance lies not in drawing a causal connection but in documenting that these radiographic confounders may be present in the background of complex pain presentations. Our results show that patients in the MVA group present, on average, 38% more RCs compared to those in the non-MVA group. Some of these conditions may have existed before the accident but were not symptomatic or noticeable until the trauma, when heightened pain awareness brought them to attention. The trauma from the MVA may also have exacerbated these conditions, further complicating diagnosis and treatment [27]. The presence of these confounders underscores the diagnostic complexity in post-MVA patients, particularly in medical-legal contexts, where determining whether an injury was caused or aggravated by the accident is critical.

Our findings that RC is more prevalently found in patients with an MVA history are worthy of attention. The diagnostic overlap between confounders and TMD symptoms in post-MVA patients underscores the complexity of evaluating orofacial pain. Impacted dentition, particularly third molars, can result in trismus and localized jaw pain, both of which are hallmarks of TMD [14]. Without distinguishing the underlying cause, these symptoms can easily be misdiagnosed as TMD. Similarly, sinus pathologies such as maxillary sinusitis can lead to midfacial pain that closely mimics the masseter muscle pain commonly associated with TMD [15]. This is particularly challenging for clinicians, as maxillary sinus inflammation can present with facial pain that overlaps the same regions where myofascial TMD symptoms manifest. Periapical pathology, including endodontic lesions, adds another layer of diagnostic complexity. These lesions can refer pain to the jaw or facial structures, mimicking the pain patterns seen in TMD [16]. If not correctly identified, this can lead to incorrect treatment plans focused on TMD rather than addressing the dental origin of the pain. In addition, an elongated stylohyoid process can contribute to neck pain, a common symptom in both whiplash-associated disorders and TMD, further blurring the lines between different pain sources [17]. Finally, patients with a history of condylar fractures may experience re-aggravation of joint pain after an MVA, complicating the differentiation between new trauma and pre-existing injury [18, 19]. These factors necessitate a comprehensive clinical and radiographic approach to accurately diagnose the true source of pain in post-MVA patients, avoiding unnecessary treatment delays and misdiagnosis of TMD. Moreover, RC may result in unwarranted delays to treatment and additional costs to the patient or their insurer. According to the 2024 Alberta Dental Fee Guide, fees charged for a clinical examination of the TMJ by a relevant specialist are estimated to be CAD (Canadian Dollar) $380–420 and the cost for a large FOV CBCT with interpretation is estimated to cost CAD $550–650 [28]. A patient who is referred to a relevant specialist for TMJ consultation and advanced imaging only to have the specialist determine that the orofacial pain post-MVA is due to a confounding entity, such as an endodontic lesion, would cause unnecessary delays and extra expenses for either the patient or the insurer.

In a study conducted by Cağlayan and Tozoğlu on a general TMD patient pool, sinus abnormalities were present in 25.9% of the patients who underwent a CBCT scan [29]. This rate is similar to our non-MVA group, but lower than our MVA cohort. They also concluded that impacted dentition and endodontic lesions accounted for 34.1% and 5.9% of the incidental findings respectively. Differences in prevalence can be possibly related to their smaller sample size (n = 85), less than a quarter of our combined sample size, and different patient demographics.

Our findings have important clinical implications, particularly in recognizing radiographic confounders (RCs) in the diagnosis and management of TMD-related pain in MVA patients. These confounders can obscure the true source of pain, leading to misdiagnoses or delays in appropriate treatment. Moreover, MVA patients often present with multiple pain complaints, which must be carefully evaluated. A MVA may exacerbate or trigger a previously asymptomatic condition, which, due to changes in pain perception, direct trauma or psychological factors (*e.g.*, stress or anxiety), may become painful [30, 31]. For instance, post-MVA patients frequently report worsening jaw parafunction, which could lead to increased pain in teeth with endodontic lesions or in patients with pre-existing sinus pathology [31]. Therefore, clinicians must remain vigilant in conducting thorough clinical examinations and considering a broad differential diagnosis before attributing facial pain solely to TMD. This comprehensive approach is crucial, particularly in complex post-MVA cases.

To explain the cause of this observed increase in RC in the MVA cohort, we have three possible hypotheses. The first theory postulates that patients who report orofacial pain after an accident may initially have more pre-existing subclinical pathologies. After a traumatic accident, patients may be more vigilant about pain and may start to notice previously asymptomatic pathology after periods of rest. It is well-documented in the scientific literature that patients report a greater severity of dental pain during nighttime when resting and a positive feedback loop exists between pain and poor sleep [32, 33]. In search for a cause, an internet search combining the terms “jaw pain” or “facial pain” and “car accident” will primarily reveal articles suggesting TMD. If the patient presents to their primary care physician or dentist complaining of orofacial pain after a MVA with a self-diagnosis of TMD, the primary clinician may immediately initiate referrals to specialists for joint evaluation without first eliminating RC. Considering that only a minority of patients develop TMJ pain following a whiplash injury [34], the increased incidence of RCs in this subset of patients may be attributable to flare-ups of previously asymptomatic entities. The second explanation for the increase in RC is related to the finding that drivers with more pre-existing chronic medical conditions are associated with a higher risk of being involved in MVA and are more likely to be deemed at fault [35, 36]. Expectantly, the presence of chronic medical conditions is associated with more dental diseases and a lack of adequate routine oral hygiene practices [37, 38, 39]. Under this circumstance, we may inadvertently select patients with more pre-existing RC by segregating the cohorts based on the history of MVA. Furthermore, it is common knowledge that old age is associated with more chronic diseases [40]. Coincidentally, the average age of the MVA cohort in the current study is 41.3 years compared to the younger average age of only 33.6 years in patients without MVA history and the data further supports this theory. Another consideration is the financial factors relating to dental treatment and the public health system burden. Unfortunately, nowadays, many people do not have insurance or the financial means to see a dentist regularly. As such, some people never see the dentist, or only do it on an emergency basis. It is estimated that 24% of Canadians avoid visiting a dental professional simply due to cost considerations [41]. However, all patients are legally required to have insurance to operate a motor vehicle. Some patients will receive financial coverage for assessment and treatment through their car insurance. At this stage, many neglected and undiagnosed dental pathologies may be found hence explaining why more RC are found in this group.

Many working hours are lost in specialist centers every year due to inappropriate or unnecessary referrals. Studies analyzing referral patterns of primary clinicians consistently revealed that up to a third of referrals can be considered to be “unnecessary” [42]. Based on limited available data, it is estimated that a considerable number of these hours could be saved if thorough clinical examinations were conducted before ordering advanced imaging like CBCT and subsequent referral. Such diagnostic rigour could prevent unnecessary appointments and radiographic testing, ultimately reducing time and financial costs for both patients and clinicians.

While imaging plays a critical role in diagnosing TMD-related pain, the importance of strong clinical diagnostic skills cannot be overstated. Clinicians must develop and apply fundamental diagnostic protocols to minimize misdiagnosis, ensuring that advanced imaging is used judiciously and only after other potential confounders have been ruled out. Comprehensive clinical evaluations are essential to making informed decisions about when advanced imaging is truly necessary.

Our study has its limitations, one of which is the lack of detailed clinical information for the 3D imaging included. Some radiographic confounders identified may be asymptomatic, which could affect the interpretation of our results. While we documented these findings, their clinical relevance may vary, particularly in the absence of symptoms. This underscores the importance of integrating timely imaging with thorough clinical evaluations to ensure that radiographic findings correspond to the patient’s clinical presentation. This is particularly critical in distinguishing symptoms directly related to motor vehicle accidents (MVAs) from pre-existing conditions or incidental findings. These considerations are especially important in medical-legal contexts, where accurate diagnosis and causality are pivotal. Furthermore, we did not have data on the severity or specifics of the MVAs. Additionally, referral forms seldom included assessments from the primary care dentist or physician or information on whether MVA cases were managed differently before referrals. The time between the MVA and the CBCT scan varied and was not considered, potentially influencing some radiographic findings and clinical presentations, though the latter was not assessed. However, our focus on pain confounders—known to persist without treatment—supports the study’s validity. The CBCT evaluations were explored retrospectively, as no formal protocol was used, which may have introduced variability in the consistency of reporting results.

For future directions, additional research on the radiographic changes in the TMJs following MVA with long-term follow-up and comparison to patients experiencing similar trauma without TMD symptoms developing can further reveal the pathophysiology of whiplash trauma to the TMJs.

Through this study, we aim to raise awareness among dentists, general physicians, and the broader medical and legal community that multiple sources of orofacial pain can mimic TMD-related pain, thereby improving the diagnosis and management of this subset of patients after an MVA. Based on our findings, we suggest that patients suspected of having TMD should undergo a thorough dental evaluation to rule out dental pathology before being referred to the appropriate dental specialist for assessment. If advanced imaging is needed after a clinical examination has been performed, the referring clinician should include all relevant clinical information in the radiographic requisition form, including the presenting signs and symptoms of TMD and details of the MVA, such as severity and timing of the incidence. We also emphasize the importance of having an OMR to complete a thorough interpretive report of the entire CBCT volume to identify radiographic confounders. These recommendations are especially critical for patients suspected of TMD following MVA, as MVA cases often involve lengthy and costly treatments and litigation.

## 5. Conclusions

Patients with a history of MVA are associated with a significantly greater number of RC (38% more per patient) revealed in their CBCT assessment of the TMJ. The most prevalent RC that can mimic TMJ-related pain included sinus pathologies and impacted dentition, which were found significantly more often in the MVA cohort. Based on these findings, we strongly recommend that all patients suspected of TMD should first undergo a general dental evaluation before being referred to the appropriate dental specialist for management or advanced imaging. This is particularly crucial for MVA patients to prevent unnecessary investigations, financial burdens and treatment delays. Awareness of these confounders is essential for patients, primary care clinicians, legal advisors and insurers.
